# Preparation and Electrocapacitive Properties of Hierarchical Porous Carbons Based on Loofah Sponge

**DOI:** 10.3390/ma9110912

**Published:** 2016-11-10

**Authors:** Zichao Li, Kuilu Zhai, Guoqiang Wang, Qun Li, Peizhi Guo

**Affiliations:** 1 State Key Laboratory Breeding Base of New Fiber Materials and Modern Textile, Institute of Materials for Energy and Environment, School of Materials Science and Engineering, Qingdao University, Qingdao 266071, China; zichaoli@qdu.edu.cn (Z.L.); 2015020673@qdu.edu.cn (G.W.); 2 School of Chemistry and Chemical Engineering, Qingdao University, Qingdao 266071, China; zhaikuilu@gmail.com; 3 Collaborative Innovation Center for Marine Biomass Fibers, Materials and Textiles of Shandong Province, Qingdao University, Qingdao 266071, China

**Keywords:** loofah sponge, porous carbon, biomass, carbonization, capacitance

## Abstract

Four porous carbon samples denoted as LSC-1, LSC-2, LCS-3, and LSC-4 were prepared by carbonization of loofah sponge pretreated by ZnCl_2_ activation, immersion in *N*,*N*-dimethylformamide (DMF), DMF-assisted solvothermal and melamine-assisted hydrothermal processes, and the specific surface areas were 1007, 799, 773, and 538 m^2^·g^−1^ with mainly micropores, respectively. Electrocapacitive properties of four porous carbon-based electrodes were investigated with cyclic voltammetry, galvanostatic charge–discharge, and electrochemical impedance spectroscopy in symmetric supercapacitors. All the cyclic voltammetries of four types of supercapacitors showed a rectangular shape, even under a high scan rate of 500 mV·s^−1^. The capacitances of LSC-1, LSC-2, LSC-3, and LSC-4 were 107.4, 92.5, 60.3, and 82.3 F·g^−1^ at the current density of 0.1 A·g^−1^, respectively, and LSC-1 displayed the excellent capacitance retention of about 81.3% with a current density up to 5 A·g^−1^. All supercapacitors showed excellent electrochemical stability, and the LSC-1-based supercapacitor showed a cycle stability with 92.6% capacitance retention after 5000 cycles at 1 A·g^−1^. The structure–property relationship of LSC samples is discussed and analyzed on the basis of the experimental data.

## 1. Introduction

Issues concerned with energy are among the most remarkable global challenges of the 21st century. The global demand for energy supply and environmental protection is increasing dramatically, forcing the world to face an economic crisis. Based on recent reports, the annual global energy consumption is about 4.1 × 10^20^ J, of which 80% comes from non-renewable carbon-intensive fossil fuels, including coal, oil, and natural gas [1,2]. Excessive reliance on the burning of fossil fuels brings along many environmental problems, leading an increased burden on the global economy. With the continuous development of the world, the demand for energy grows rapidly while the total non-renewable energy sources of the Earth are rather limited. Therefore, developing energy conversion and storage devices with safety, practicability, sustainability, and good-performance is imperative in order to meet the growing energy demand and reduce environmental stresses [3,4,5].

Electrochemical capacitors have aroused much interest in researchers recently, due to their numerous advantages, including remarkable cycle efficiency, desired cycle life, fast charge/discharge rate, and excellent power density, as well as their potential applications in portable electronics, hybrid electric vehicles, and memory back-up equipment [6,7,8]. It is beyond doubt that the performance of electrochemical double layer capacitors is largely governed by the types and forms of their electrode materials. Studies of potential electrode materials for supercapacitors based on various forms, functions, and textures of porous carbon have been reported [9,10,11,12,13,14]. In the view of electrode materials for supercapacitors, intensive attention has been paid to carbon materials, such as activated carbon, graphene, carbon nanostructures like tubes and fibers, carbide-derived carbon, and so on [15,16,17,18]. In addition, easily available and renewable carbon materials with the advantages of more plentiful porous structure, heteroatom self-doping, great physicochemical stability, and being environmentally friendly are widely applied in the energy-related fields to meet the urgent demand of energy supply [19,20,21,22]. Up to now, a variety of biomass materials have been vastly used as the precursors to obtain char materials for capacitors, like fruits [23,24], nutshells [25,26,27], grains [28,29], seaweeds [30], animal bone [31], fungi [32], etc. [33,34,35,36,37]. Most of these reports claimed that the energy storage performance of biomass-derived carbons approximated—or even surpassed—that of the commercial activated carbon.

Loofah sponge—as an easily accessible vegetal resource in China—is one of the renewable and sustainable biomass materials with a natural porous network. Making full use of loofah sponge is still a challenge, although it has been extensively utilized as adsorbent [38], cell immobilization medium [39], and composite materials [40]. In this paper, hierarchical porous carbon materials—of which the main structure is micropore—were obtained via the carbonization of loofah sponge pretreated by various strategies, including ZnCl_2_ activation, *N*,*N*-dimethylformamide (DMF)-based process, and melamine-assisted hydrothermal process. Results showed that the porous carbons obtained by using ZnCl_2_ activation had the largest specific surface area among the four carbon samples, and the highest specific capacitance of 107.4 F·g^−1^ at 0.1 A·g^−1^ in symmetrical supercapacitors with 6 M KOH solutions as the electrolyte. Furthermore, all of the porous carbons exhibited good electrochemical stability, even after 5000 cycles. In the following, the structure–property relationship of the loofah sponge-based porous carbon (LSC) samples is discussed and analyzed on the basis of the experimental data.

## 2. Materials and Methods

### 2.1. Reagents and Materials

The loofah sponge was collected from a nearby supermarket. Most chemicals, such as NaOH, KOH, iso-propyl alcohol and ethylene glycol were purchased from Sinopharm Chemical Reagent Corp. (Shanghai, China). DMF and melamine were obtained from Tianjin Kermal Chemical Corp. (Tianjin, China). Polytetrafluoroethylene latex (PTFE, 20 wt %) and acetylene carbon black (99.99%) were purchased from Sigma-Aldrich Chemie GmbH (Deisenhofen, Germany) and Strem Chemicals, Inc. (Boston, MA, USA), respectively. Double-distilled water and analytical grade reagents were used without further purification in all experiments.

### 2.2. Preparation of the Loofah Sponge-Based Porous Carbons (LSCs)

Pretreatment of loofah sponge: loofah sponge was shattered by pulverizer and then pretreated in 30% sodium hydroxide solution at 80 °C for 2 h. The treated loofah sponge was thoroughly washed with distilled water and placed in an oven at 80 °C for drying.

Preparation of ZnCl_2_ activated porous carbon (LSC-1): 2 g of dried loofah sponge was preheated up to 400 °C at a heating rate of 5 °C·min^−1^ and kept at this temperature for 2 h, then cooled to room temperature naturally. The pre-carbonized solid product and ZnCl_2_ were completely mixed in an agate mortar at a weight ratio of 13/1 (solid product/ZnCl_2_), followed by heating up to 800 °C in a horizontal furnace at a heating ramp rate of 5 °C·min^−1^ and then holding for 2 h under Argon gas flow. The obtained powders were distilled with water and then dried in an oven.

Porous carbon prepared from loofah sponge after immersing in DMF (LSC-2): 2 g of loofah sponge was immersed in 16 mL DMF for 24 h, then dried and heated to 800 °C in a horizontal furnace at a heating ramp rate of 5 °C·min^−1^ under Argon gas flow and kept at 800 °C for 2 h, then cooled to room temperature naturally.

Porous carbon prepared from solvothermal-treated loofah sponge (LSC-3): after 2 g of loofah sponge was added into 20 mL Teflon lined stainless autoclaves, 16 mL of DMF were added into the autoclaves. The reactions proceeded at 200 °C for 6 h and then naturally cooled to room temperature, followed by filtering for sample collection, washing with abundant water, and then drying at 75 °C overnight. Finally, samples were calcinated at 800 °C for 2 h with a heating rate of 5 °C·min^−1^.

Porous carbon prepared from hydrothermal-treated loofah sponge (LSC-4): 0.86 g of melamine powder was dissolved in 10 mL distilled water, and the solutions were added into the autoclaves. The reactions were proceeded at 200 °C for 6 h, and then naturally cooled to room temperature, followed by filtering for collection of the samples, washing with abundant water, and then drying at 75 °C overnight. Finally, samples were calcinated at 800 °C for 2 h with a heating rate of 5 °C·min^−1^.

### 2.3. Characterization of the Porous Carbon

X-ray powder diffraction (XRD) was conducted by using an X-ray diffractometer (D8 Advance, Bruker AXS GmbH, Karlsruhe, Germany) equipped with Cu Kα radiation (λ = 0.15418 nm). The pore structures of the samples were measured by physical adsorption of nitrogen at liquid nitrogen temperature (77 K) on an automatic volumetric sorption analyzer (NOVA 1100, Quantachrome Corp., Boynton Beach, FL, USA). The specific surface area of the samples was determined by the Brunauer–Emmett–Teller (BET) method. Pore size distribution was evaluated by the Barrett–Joyner–Halenda (BJH) method. Fourier-transform infrared spectra (FT-IR) were recorded on an infrared spectrophotometer (Nicolet 5700, Thermo Fisher Scientific Inc., Waltham, MA, USA) using KBr pellets.

### 2.4. Electrochemical Performance

The electrodes were composed of 5 wt % PTFE, 10 wt % acetylene carbon black, and 85 wt % porous carbon active materials [35,41]. All materials were mixed uniformly and then pressed onto a nickel foam substrate at 1.4 MPa, followed by drying in a vacuum oven at 110 °C for 12 h. The amount of active materials on each current collector was about 4.7 mg·cm^−2^. Symmetrical two-electrode cells were assembled with aqueous 6 M KOH electrolyte isolated by a porous membrane. Cyclic voltammetry (CV), galvanostatic charge–discharge (GCD) and electrochemical impedance spectroscopy (EIS) tests were employed to study the capacitive performance of the samples. The EIS spectra were measured at open circuit potential in the frequency range of 0.01 Hz–100 KHz with the amplitude of 5 mV. All the electrochemical measurements were performed on an electrochemical workstation (CHI760e, CH Instrument Inc., Austin, TX, USA). All the tests were conducted at room temperature.

## 3. Results and Discussion

### 3.1. Morphology and Structure of Porous Carbons

**Figure 1:**
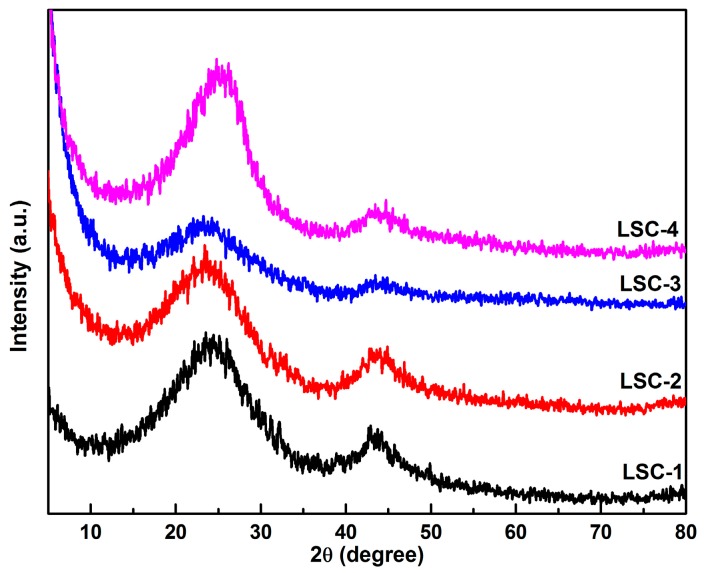
X-ray powder diffraction (XRD) patterns of loofah sponge-based porous carbon (LSC) samples.

The XRD patterns of the four porous carbon samples reveal the absence of sharp and strong peaks (as shown in Figure 1), indicating the amorphous state of all the LSC samples. On one hand, one broad peak located at about 23° and a weak peak located at 44° were the characteristic peaks of amorphous graphitic carbon, suggesting a limited degree of graphitization of LSC samples. On the other hand, the appearance of the weak peak centered at 44° indicated the improvement of the electrical conductivity. Especially, a slightly sharper peak observed at 44° for LSC-1 suggested the enhanced graphitization degree of LSC-1 compared with the other samples.

**Figure 2:**
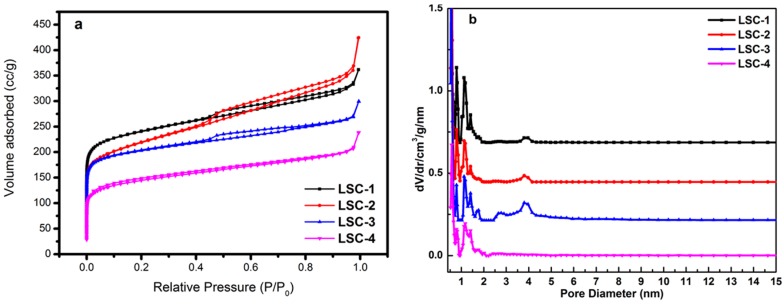
(**a**) Nitrogen adsorption–desorption isotherms and (**b**) pore size distributions of of LSC samples.

The porosity properties of carbon materials were analyzed by nitrogen adsorption and desorption measurements. The nitrogen adsorption–desorption isotherms and the pore size distribution of all the LSC samples are shown in Figure 2. These carbon materials have type I and IV isotherms with different BET specific surface areas. The specific surface areas of the samples were found to be 1007 m^2^·g^−1^ for LSC-1, 799 m^2^·g^−1^ for LSC-2, 773 m^2^·g^−1^ for LSC-3, and 538 m^2^·g^−1^ for LSC-4, as calculated from nitrogen isotherms at −196.6 °C. Obviously, a large amount of absorbed nitrogen at the low relative pressure was observed, which is characteristic of microporous materials. A hysteresis loop with the value of P/P_0_ extending from 0.42 to 0.95 can be observed for LSC-1, LSC-2, and LSC-3, reflecting the coexistence of both the micropore and mesopore structures in these materials. The pore-size distribution gained from the isotherm suggests that a great quantity of pores less than 5 nm exists in LSC-1, LSC-2, and LSC-3. However, LSC-4 is typical for microporous materials. These pores are more likely to arise from the spaces among the numerous pore structures themselves within the LSC samples. The sharp distribution of micropores around 0.6 nm indicates that the LSC samples have distinct potential for energy storage. The single-point total volume of pores for LSC-1 at P/P_0_ = 0.99512 was 0.438 cm^−3^·g^−1^. These results suggest that the ZnCl_2_ activated carbonization method endowed the LSC with the largest specific surface area. However, either DMF immersion or DMF-assisted solvothermal process could not lead to a drastic difference in the physical properties of LSCs. LSC-4 pretreated by melamine-assisted hydrothermal process showed the smallest specific surface area with only microporous structure, probably because of the complicated reaction during the process. More detailed structural information is listed in Table 1.

### 3.2. Electrochemical Characterization of LSC

**Figure 3:**
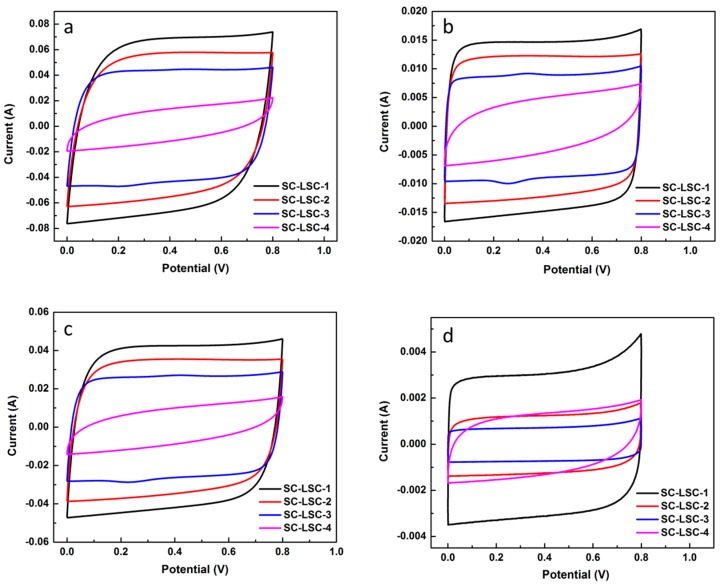
Cyclic voltammetry (CV) curves of LSC-based supercapacitors (SC-LSCs) at different scan rates (mV·s^−1^): (**a**) 20; (**b**) 100; (**c**) 300; and (**d**) 500.

The electrocapacitive properties of LSC-based supercapacitors (SC-LSCs)—denoted as SC-LSC-1, SC-LSC-2, SC-LSC-3, and SC-LSC-4, assembled from LSC-1, LSC-2, LSC-3, and LSC-4, respectively—were evaluated by a sequence of electrochemical techniques, including CV, GCD, and EIS tests at room temperature. Figure 3 represents the CV curves of four SC-LSCs at different scan rates with a potential window ranging from 0 to 0.8 V. As seen, all of the SC-LSCs exhibit an ideal capacitive behavior with rectangular shape at relatively low scan rates, namely the typical nature of double-layer capacitor during the charge–discharge process. Even at a high scan rate of 500 mV·s^−1^, as shown in Figure 3d, the CV profiles of SC-LSCs maintain the quasi-rectangular shape of the voltammograms with little distortion, which can be ascribed to the excellent electrical conductivity and the low mass-transport resistance of the samples. Apparently, the rapid ionic transportation due to the short ion pathway and electrolyte reservoir are derived from the hierarchical pore structure of LSC samples. Similarly, compared with other samples, SC-LSC-1 shows the highest capacitance value due to the ideal hierarchical porous structure and high surface area of LSC-1.

**Figure 4:**
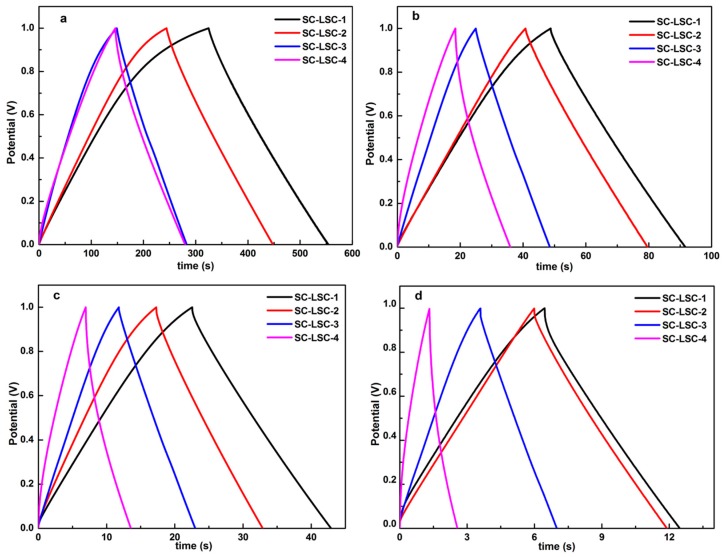
Galvanostatic charge–discharge (GCD) curves of SC-LSCs at different current densities (A·g^−1^): (**a**) 0.1; (**b**) 0.5; (**c**) 1; and (**d**) 3.

The GCD curves of all the SC-LSCs are shown in Figure 4, the electrodes of which show a typical triangular shape, although imperfect symmetry with a small IR drop. According to the discharging profiles (Figure 4a), based on the charge–discharge curves, the specific capacitance of LSC samples can be calculated by the following formula:

C = IΔt/ΔVm

where C, I, Δt, ΔV, and m are the specific capacitance (F·g^−1^), the discharge current (A), the discharge time (s), the voltage range (V), and mass of active material (mg). The capacitances of LSC samples were measured to be about 107.4, 92.5, 60.3, and 82.3 F·g^−1^ at the current density of 0.1 A·g^−1^ for LSC-1, LSC-2, LSC-3, and LSC-4, respectively. When the current density was 1 A·g^−1^ (Figure 4c), the capacitances of SC-LSCs were 87.8, 81.8, 49.3, and 68.1 F·g^−1^ for LSC-1, LSC-2, LSC-3, and LSC-4, respectively. Then, the corresponding capacitances slightly decreased to 82.0, 79.5, 45.6, and 57.2 F·g^−1^ with an increase in the current density up to 3 A·g^−1^ (Figure 4d). A large decrease in the capacitance of SC-LSCs was seen at low current densities, followed by slight increase in the high current density. It can be concluded from all the GCD curves in Figure 4 that the discharge time for SC-LSC-4 was obviously decreased compared with the other three supercapacitors.

**Figure 5:**
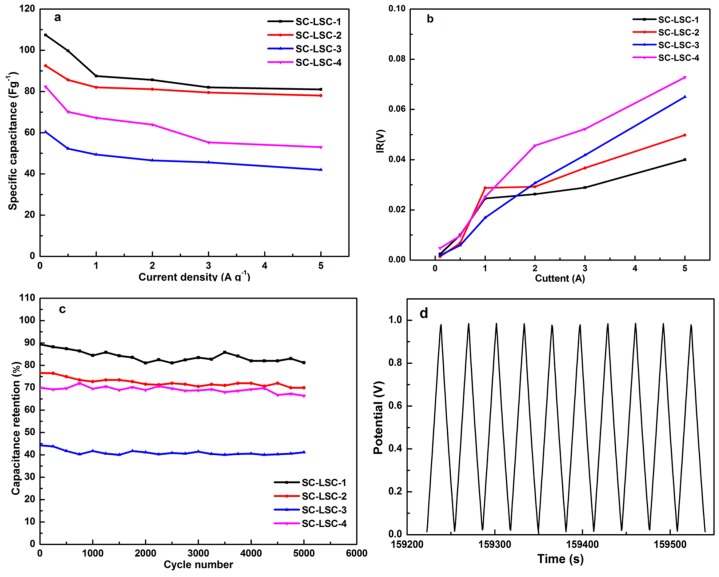
(**a**) Capacitive curves of SC-LSCs at different current densities; (**b**) Inner resistance variations as a function of the current density in SC-LSCs; (**c**) cycle stability of all the SC-LSCs at 1 A·g^−1^ over 5000 cycles; and (**d**) the last 10 charge/discharge curves of SC-LSC-1 at 1 A·g^−1^ over 5000 cycles.

The variations of the capacitance with the current density of SC-LSCs are shown in Figure 5a. The capacitances of SC-LSCs decreased obviously when the current density was less than 1 A·g^−1^. With the continuous increase of the current density, the capacitances were almost constant, especially for SC-LSC-3 and SC-LSC-4. As depicted in Figure 5b, the variations of inner resistance (IR) drop with the current density of all the SC-LSCs suggests the low values of less than 0.03 V for IR drop when the current density was no more than 1 A·g^−1^. A slight increase of IR drop was observed with a further increase of current density. The smallest IR drop at 5 A·g^−1^ was less than 0.04 V for SC-LSC-1. Even for SC-LSC-4, the largest IR drop at 5 A·g^−1^ was only about 0.075 V. The cycling stability of these symmetric supercapacitors was evaluated at the current density of 1 A·g^−1^. As derived from Figure 5c, even undergoing 5000 charge–discharge cycles, there is still above 92% capacitance retention for all the SC-LSCs, indicating the prominent electrochemical stability of LSC-based supercapacitors. The last 10 charge/discharge curves (4991th–5000th cycles) for SC-LSC-1 are shown in Figure 4d. It can be obviously seen that all the GCD curves show almost symmetrical shapes. The capacitance calculated from the final cycles is about 82 F·g^−1^ at 1 A·g^−1^ for SC-LSC-1—92.6% of the initial value. These results indicate that loofah sponge can be used as carbon precursor to prepare porous carbons with a good potential for electrode materials. The excellent capacitance retention of SC-LSCs during the cycle test should be ascribed to its highest specific surface area with proper microporous and mesoporous structures.

**Figure 6:**
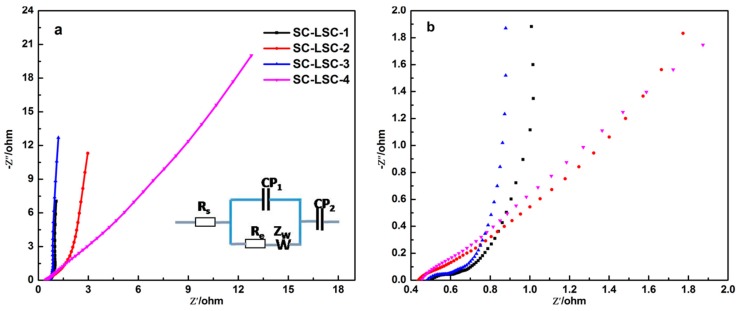
(**a**) Nyquist plots of four types of SC-LSCs in the frequency range of 0.01 Hz to 100 KHz under open circuit potential conditions and (**b**) the magnified profile. The inset in (**a**) is the equivalent circuit of Nyquist plots.

For supercapacitors, the relevant frequency behavior and equivalent series resistance (ESR) are usually assessed with the powerful tool of EIS [41,42]. For the purpose of learning more about the electrodes with remarkable power performance, the Nyquist plots (Figure 6a) of the SC-LSCs were characterized in a frequency ranging from 0.01 Hz to 100 KHz. According to the enlarged plot curves shown in Figure 6b, a semicircular shape was seen at high frequency, while a drastic increase in the imaginary part of the impedance was seen at a lower frequency. In the high frequency range, the left plot intersection at the real part (Z’) is associated with the electrolyte resistance (R_s_) and the square resistance of all the SC-LSCs. The semicircle region of the plot curve for each SC-LSC accounts for the charge transfer resistance (R_c_), which indicates the migration rate of hydrated K^+^ and OH^−^ ions in SC-LSCs at the interface between the solution and the electrode surface.

Furthermore, the Warburg resistance (Z_w_) section—in which region the slope of the plots is about 45°—is very short for all of the SC-LSCs, indicating an effective access of the K^+^ or OH^−^ ions and a desired short diffusion pathway to the LSC electrode surface. The greater the slope of the low frequency line, the closer to the ideal supercapacitor and electrocapacitive behavior [39]. At the same time, the smaller arc diameter in the EIS spectrum of SC-LSC-1 indicates lower charge-transfer resistance. The slopes of both SC-LSC-1 and SC-LSC-3 approach an ideally straight line, implying the enhanced accessibility of the ions.

**Figure 7:**
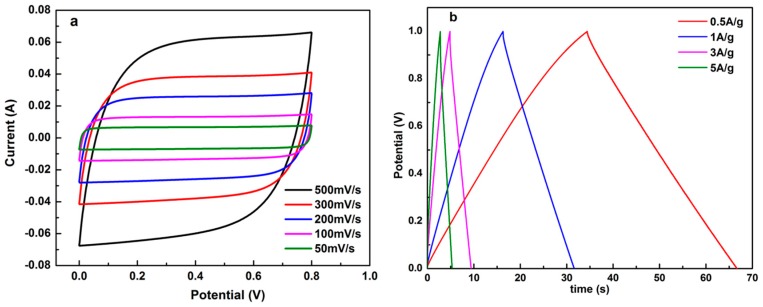
(**a**) CV and (**b**) GCD curves of SC-LSC-1 (2 M KOH) under different conditions.

It was reported that the concentration of the electrolytes in supercapacitors can affect their electrochemical performance [43,44]. Usually, the higher the electrolyte concentration in SC-LSCs, the faster the movement of the hydrated ions during the electrochemical measurement. When the concentration of KOH in the electrolytes decreased to 2 M (as shown in Figure 7), the electrochemical properties of the LSC-SCs were obviously different from those based on 6 M KOH solutions. Firstly, taking LSC-1 as an example, the shape of the CV curve of SC-LSC-1 (2 M KOH) was quasi-rectangular and somewhat tilted (as illustrated in Figure 7a), compared with that of SC-LSC-1 at the same scan rate. Secondly, the capacitance of SC-LSC-1 (2 M KOH) was obviously lower than that of SC-LSC-1. For example, the capacitances of SC-LSC-1 (2 M KOH) decreased to 91.0, 87.0, 80.5, and 75.6 F·g^−1^ at the current densities of 0.5 A·g^−1^, 1 A·g^−1^, 3 A·g^−1^, and 5 A·g^−1^ (Figure 7b), respectively, which only represented about 91.3%, 97.3%, 98.3%, and 93.8% of those of SC-LSC-1.

## 4. Conclusions

Four types of porous carbons were synthesized from loofah sponge based on different pretreatments before carbonization. All prepared loofah sponge-based porous carbons showed mainly microporous structure. It was found that porous carbons prepared by ZnCl_2_ activation method showed the largest specific surface area and displayed the best electrochemical performance among all the loofah sponge-based porous carbon samples, with a capacitance of 107.4 F·g^−1^ at 0.1 A·g^−1^ in symmetrical two-electrode cells. The capacitances of these porous carbon-based supercapacitors only changed slightly with current densities larger than 1 A·g^−1^. All of the supercapacitors showed excellent electrochemical stability, and the capacitance of the LSC-1-based supercapacitor maintained above 92.6% after 5000 charge–discharge cycles at 1 A·g^−1^. These results indicate that loofah sponge can be considered as a good candidate to prepare hierarchical porous carbons with high surface area and excellent electrochemical properties through rational design.

## Figures and Tables

**Table 1:** Specific surface areas, pore volumes, and pore width of LSC samples. S_BET_: Brunauer–Emmett–Teller surface area; DMF: *N*,*N*-dimethyl formamide.

Sample	Preparation Method	S_BET_ (m^2^·g^−1^)	Micropore Area (m^2^·g^−1^)	MicroporeVolume (cm^−3^·g^−1^)	V_pore_ (cm^−3^·g^−1^)	Pore Width (nm)
LSC-1	ZnCl_2_ activation	1007	753	0.273	0.438	0.545
LSC-2	DMF soaking	799	459	0.191	0.444	0.548
LSC-3	DMF solvothermal treatment	773	608	0.243	0.328	0.573
LSC-4	Melamine hydrothermal treatment	538	382	0.156	0.258	0.578
